# Relação entre Gordura Epicárdica e Fibrilação Atrial Não Pode Ser Totalmente Explicada pela Fibrose Atrial Esquerda

**DOI:** 10.36660/abc.20201083

**Published:** 2022-01-11

**Authors:** Daniel Matos, António Miguel Ferreira, Pedro Freitas, Gustavo Rodrigues, João Carmo, Francisco Costa, João Abecasis, Pedro Carmo, Carla Saraiva, Diogo Cavaco, Francisco Morgado, Miguel Mendes, Pedro Adragao

**Affiliations:** 1 Centro Hospitalar de Lisboa Ocidental EPE Hospital de Santa Cruz Lisboa Portugal Centro Hospitalar de Lisboa Ocidental EPE Hospital de Santa Cruz, Lisboa – Portugal

**Keywords:** Fibrilação Atrial, Fibrose Atrial, Gordura Epicárdica, Isolamento da Veia Pulmonar

## Abstract

**Fundamento:**

O tecido adiposo epicárdico (TAE) tem sido associado à fibrilação atrial (FA), mas seus mecanismos fisiopatológicos permanecem obscuros.

**Objetivos:**

Medir a correlação entre TAE e fibrose do átrio esquerdo (AE), e avaliar sua capacidade de prever recidiva após o isolamento da veia pulmonar (IVP).

**Métodos:**

Pacientes com FA inscritos para um primeiro procedimento de IVP foram submetidos à tomografia computadorizada (TC) cardíaca e ressonância magnética cardíaca (RMC) em menos de 48 horas. Quantificou-se o TAE_CE_ em imagens de TC realçadas com contraste no nível do tronco da coronária esquerda. Quantificou-se a fibrose do AE em RMC tridimensional com realce tardio isotrópico de 1,5 mm. Após o isolamento da veia pulmonar (IVP), os pacientes foram submetidos a seguimento para checar a recidiva da FA. A significância estatística foi definida com p<0,05.

**Resultados:**

A maioria dos 68 pacientes (46 homens, idade 61±12 anos) tinha FA paroxística (71%, n=48). Os pacientes apresentavam volume TAE_CE_ mediano de 2,4 cm^3^/m^2^ (intervalo interquartil [IIQ] 1,6–3,2 cm^3^/m^2^) e um volume médio de fibrose do AE de 8,9 g (IIQ 5–15 g). A correlação entre TAE_CE_ e fibrose do AE foi estatisticamente significativa, mas fraca (coeficiente de correlação de postos de Spearman = 0,40, p=0,001). Durante um seguimento médio de 22 meses (IIQ 12–31), 31 pacientes (46%) tiveram recidiva da FA. A análise multivariada produziu dois preditores independentes de recidiva da FA: TAE_CE_ (FC 2,05, IC de 95% 1,51–2,79, p<0,001) e FA não paroxística (FC 2,36, IC de 95% 1,08–5,16, p=0,031).

**Conclusão:**

A correlação fraca entre TAE e AE sugere que a fibrose do AE não é o principal mecanismo que liga o TAE e a FA. O TAE mostrou-se mais fortemente associado à recidiva da FA do que à fibrose do AE, corroborando a existência de outros mediadores mais importantes do TAE e da FA.

## Introdução

Recentemente, demonstrou-se que o tecido adiposo epicárdico (TAE) esteve associado à presença, gravidade e reincidência da fibrilação atrial (FA).^1^ Embora os mecanismos fisiopatológicos subjacentes a essa associação ainda não tenham sido estabelecidos, foram levantadas diversas hipóteses, incluindo infiltração direta de adipócitos, estresse oxidativo e secreção de adipocinas, causando inflamação e fibrose do tecido atrial.^1^ Pode ser útil determinar se essa relação é causal e verificar seus processos subjacentes para entender melhor a FA e identificar possíveis alvos terapêuticos. Até o momento, as evidências que ligam o TAE e a fibrose atrial vieram principalmente de análises histológicas e bioquímicas de amostras obtidas em cirurgia cardíaca,^2^ mas ambas essas características podem ser avaliadas de forma não invasiva. Neste estudo, nosso objetivo foi medir a correlação entre o volume de TAE e a quantidade de fibrose do átrio esquerdo (AE) avaliada por imagem não invasiva, e avaliar sua capacidade de predizer o tempo de recidiva após o isolamento da veia pulmonar (IVP).

## Métodos

### População do estudo

Todos os pacientes consecutivos com FA sintomática refratária a medicamentos, submetidos à tomografia computadorizada (TC) cardíaca antes do IVP percutâneo no Hospital Santa Cruz (Carnaxide, Portugal) entre novembro de 2015 e dezembro de 2017, que foram submetidos à TC cardíaca e ressonância magnética cardíaca (RMC) em menos de 48 horas foram incluídos em um registro observacional utilizado para este estudo retrospectivo. Pacientes com doença cardíaca valvar moderada ou grave, trombo de átrio esquerdo, função tireoidiana anormal ou contraindicação para anticoagulação foram excluídos. A fibrilação atrial foi categorizada como paroxística se autoterminada em menos de 7 dias, persistente caso os episódios tenham durado ≥7 dias ou exigiram cardioversão, ou persistente de longa duração caso a FA tenha sido mantida por mais de 12 meses. O presente registro observacional está em conformidade com as diretrizes éticas da declaração de Helsinque e foi aprovado pelo comitê de revisão institucional. Todos os pacientes assinaram o Termo de Consentimento Livre e Esclarecido.

### Protocolos de TC e CMR cardíaca

Todos os pacientes foram submetidos a TC e cardíaca RMC menos de 72 horas antes do procedimento de ablação para avaliação da anatomia da veia pulmonar, medição do volume AE, exclusão de trombos e integração com mapeamento eletroanatômico.

As tomografias foram realizadas em equipamento com 64 cortes de fonte dupla (Somatom Definition^®^, Siemens Healthineers^®^, Erlangen, Alemanha) com injeção de 90 mL de meio de contraste não iônico (400 mg I/mL de iomeprol Bracco^®^) a uma vazão de 5 mL/s seguido por 30–50 mL de solução salina. Os parâmetros de varredura incluíram colimação do detector de 2x32x0,6 mm, aquisição de corte de 64x0,6 mm, tempo de rotação do *gantry* de 330 ms, controle automático de exposição e potencial do tubo de 100 kV (exceto se o índice de massa corporal fosse maior que 30 kg/m^2^ e o peso corporal fosse superior a 90 kg, onde 120 kV foram usados).

Utilizou-se sistematicamente a modulação prospectiva da corrente do tubo para minimizar a exposição à radiação. Realizou-se reconstrução da imagem com espessura de corte de 0,75 mm.

As imagens foram adquiridas em equipamento de 1,5 T (Magnetom Avanto^®^, Siemens Healthineers). O protocolo de varredura incluiu uma sequência isotrópica de inversão-recuperação 3D de 1,5 mm com gradiente eco *recalled* e saturação de gordura e navegador respiratório, adquirida 15 a 20 minutos após a administração de 0,2 mmol/kg de gadobutrol intravenoso. O tempo de inversão foi escolhido individualmente para anular o miocárdio normal, usando uma sequência *scout* para o tempo de inversão.

Utilizou-se o sincronismo do ECG para definir o tempo de aquisição da imagem até o final da sístole ventricular (diástole AE).

### Análise das imagens

Realizou-se a quantificação tomográfica do TAE de forma semiautomática em imagens axiais em uma estação de trabalho TeraRecon Aquarius^®^ (versão 4.4.12, TeraRecon^®^, San Mateo, CA, EUA). Quatro cortes contíguos centrados no óstio do tronco da artéria coronária esquerda (CE) foram selecionados para análise. O pericárdio foi traçado manualmente na primeira e última imagens, e interpolado automaticamente nos dois cortes do meio, que foram verificados quanto à precisão e ajustados, se necessário. Definiu-se o volume do TAE^CE^ como o volume total de tecido no interior do saco pericárdico nesta região de interesse, de 4 cortes, com valores de atenuação entre -250 e -30 unidades de Hounsfield.^1^ Calculou-se o volume atrial esquerdo traçando-se as bordas do AE nas imagens tomográficas, excluindo-se as veias pulmonares e o apêndice atrial esquerdo.^3^

**Figura 1:**
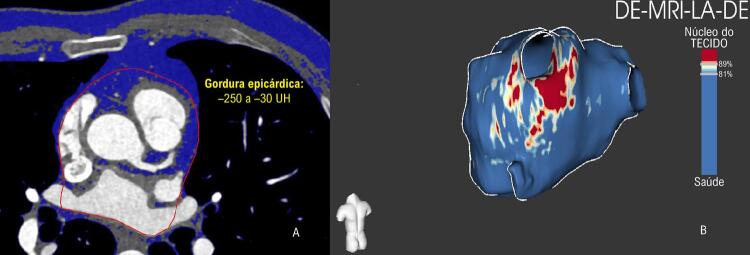
– *Gordura epicárdica (A) medida com tomografia computadorizada e fibrose de átrio esquerdo (B) medida com ressonância magnética cardíaca.*

Realizou-se o pós-processamento da RMC para quantificação da fibrose do AE com o software ADAS^®^ (versão 2.3.3, Galgo Medical). Os contornos da parede do AE foram desenhados manualmente, excluindo-se da análise a valva mitral e as veias pulmonares. A intensidade do sinal da parede do AE foi normalizada usando uma razão de intensidade de imagem (RII) calculada como a razão entre a intensidade do sinal de cada pixel e a intensidade média do pool sanguíneo. Considerou-se uma RII >1,20 como representando fibrose do AE.^4^ As quantificações de fibrose do AE e TAE foram realizadas somente após o procedimento de ablação (sem saber seu desfecho). A Figura 1 apresenta exemplos de quantificações de fibrose de TAE e AE.

### Protocolo de isolamento de veias pulmonares

O isolamento das veias pulmonares foi guiado por mapeamento eletroanatômico, utilizando os sistemas NavX^®^ (St Jude Medical^®^, St Paul, MN, EUA) ou CARTO^®^ (Biosense Webster^®^, Diamond Bar, CA, EUA). A veia femoral direita foi utilizada como acesso vascular preferencial, por meio da qual foram introduzidos três cateteres eletrodos: (i) um cateter decapolar, introduzido pelo seio coronário; (ii) um cateter de mapeamento circular variável, inserido nas veias pulmonares; e (iii) um cateter de ablação irrigado com sensor de força de contato. O acesso atrial esquerdo foi estabelecido por punção transeptal. A ablação por radiofrequência foi realizada a mais de 5 mm dos óstios das veias pulmonares, com lesões contínuas envolvendo os pares esquerdo e direito das veias pulmonares.^5^

O tratamento era considerado bem-sucedido se o bloqueio bidirecional fosse obtido. Quando necessário, realizava-se cardioversão elétrica ao final do procedimento. A anticoagulação oral foi reiniciada 6 horas após a ablação, mantida por 6 meses e então retirada ou continuada de acordo com os critérios do escore CHA_2_DS_2_-VASc. Antiarrítmicos de classe I/III foram, em geral, mantidos em todos os pacientes durante os primeiros 3 meses após o procedimento e, em seguida, retirados se não houvesse recidiva da FA. Foram prescritos inibidores da bomba de prótons no primeiro mês após a ablação.

### Desfecho do estudo e seguimento dos pacientes

O desfecho do estudo foi a recidiva da FA, definida como FA sintomática ou documentada e/ou outras arritmias atriais, após um período de supressão de 3 meses. Definiu-se FA sintomática como a presença de sintomas provavelmente em virtude de episódios de FA. Definiu-se FA documentada pela presença de pelo menos um episódio de FA com duração superior a 30 segundos em ecocardiografia, Holter de 24 horas ou monitor de eventos. O protocolo de seguimento era composto por consultas ambulatoriais com ECG de 12 derivações e Holter de 24 horas, a critério dos médicos assistentes (normalmente em 6 e 12 meses e, posteriormente, em uma base anual). Caso o prontuário fosse insuficiente, realizava-se entrevista estruturada por telefone. Os pacientes mantidos em uso de antiarrítmicos após o terceiro mês de seguimento não foram considerados como falha na ablação.

### Análise estatística

As variáveis contínuas com distribuição normal e não normal foram expressas como média±desvio padrão e mediana e intervalo interquartil, respectivamente, e as variáveis categóricas foram expressas como frequências e porcentagens. A significância estatística foi definida com p<0,05. Utilizou-se o teste de Shapiro-Wilk para avaliar a normalidade da população. Utilizou-se o teste t de Student não pareado para avaliar diferenças estatisticamente significativas entre variáveis contínuas com distribuição normal, e o teste U de Mann-Whitney para variáveis contínuas sem distribuição normal. Utilizou-se o teste do qui-quadrado para analisar as variáveis categóricas. Utilizou-se o coeficiente de correlação de Spearman para medir a correlação entre o volume do TAE_CE_ e a fibrose do AE. Utilizou-se o modelo de regressão de Cox univariada de riscos proporcionais para identificar preditores de tempo até a recidiva da FA. Variáveis com valor de p≤0,10 na análise univariada foram selecionadas para um modelo de regressão de Cox multivariado, sendo consideradas estatisticamente significativas se p<0,05. Utilizou-se um nível de confiança de 95% em nossa análise estatística. Realizou-se a análise estatística com o programa Statistical Package for Social Sciences (SPSS) versão 22.0 (SPSS Inc., Chicago, Illinois).

## Resultados

A tabela 1 apresenta as características basais da população estudada. No geral, os pacientes tiveram um volume de TAE_CE_ mediano de 2,4 cm^3^/m^2^ (intervalo interquartil (IIQ) 1,6–3,2 cm^3^/m^2^) , e um volume médio estimada de fibrose de AE de 8,9 g (IIQ 5–15 g), correspondendo a 8% (IIQ 5–11%) da massa total da parede do AE.

**Tabela 1:** – Características basais da população estudada

Características basais	Total (n=68)	Sem recidiva de FA (n=37)	Com recidiva de FA (n=31)	Valor de p
Idade, anos	61±12	61±11	61±12	0,968
Sexo masculino, n (%)	46 (67,6)	22	24	0,312
Peso, kg	81±13	79±12	82±13	0,097
Índice de massa corporal, kg/m^2^	28±4	27±4	29±4	0,091
Tipo de FA				0,003
paroxística, n (%)	48 (70,6)	32	16	
não paroxística, n (%)	20 (29,4)	5	15	
Hipertensão, n (%)	41 (60,3)	21	20	0,621
Diabetes, n (%)	6 (8,8)	5	1	0,209
Tabagismo ativo, n (%)	4 (5,9)	1	3	0,304
Disfunção sistólica do VE, n (%)	0 (0)	0	0	1,000
DAC conhecida, n (%)	6 (8,8)	4	2	0,366
CHA_2_DS_2_-VASc, mediana (IIQ)	2 (1–3)	2 (1–3)	2 (1–3)	0,578
Volume do AE na TC cardíaca, mL/m^2^	56±15	52±12	60±17	0,025
Volume do TAE_CE_, mL/m^2^	2.4±1,2	1.9±0,7	3.1±1,2	<0,001
Fibrose de AE, g, mediana (IIQ)	8,9 (5–15)	6,7 (4–13)	11,2 (6–17)	0,049
Fibrose do AE, % da massa do AE, mediana (IIQ)	7,5 (5–11)	6,9 (4–11)	8,8 (6–12)	0,170

*FA: fibrilação atrial; VE: ventrículo esquerdo; E: átrio esquerdo; TC: tomografia computadorizada; TAE_CE_: tecido adiposo epicárdico.*

**Figura 2:**
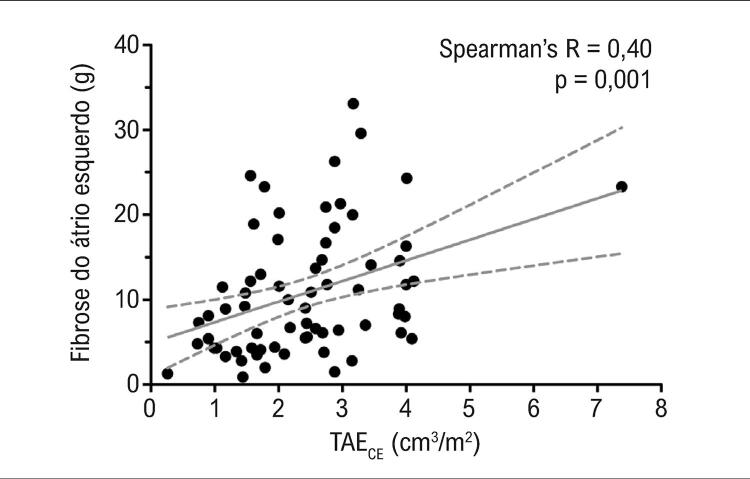
– *Gráfico de correlação de fibrose atrial esquerda e gordura epicárdica. TAE_CE_: tecido adiposo epicárdico.*

A correlação entre o TAE_CE_ e fibrose do AE foi estatisticamente significativa, mas fraca (coeficiente de correlação de Spearman = 0,40, p=0,001) — Figura 2 .

Durante um seguimento médio de 22 meses (IIQ 12–31), 31 pacientes (46%) tiveram recidiva da FA. Os pacientes que tiveram recidiva da FA eram mais propensos a ter FA não paroxística e tinham maiores volumes de AE, maiores volumes de TAE_CE_ e fibrose de AE. Ao avaliar o tempo para recidiva da FA, esses quatro preditores foram identificados na regressão de Cox univariada. A análise multivariada produziu dois preditores independentes de tempo para recidiva da FA: TAE_CE_ e FA não paroxística ( Tabela 2 ).

**Tabela 2:** – Regressão de Cox univariada e multivariada de preditores de recidiva de FA

Preditores de recidiva de FA	Análise univariada			Análise multivariada		
	RR	IC 95%	Valor de p	RR	IC 95%	Valor de p
TAE_CE_	2,19	1,65–2,91	<0,001	2,05	1,51–2,79	<0,001
FA não paroxística	3,36	1,64–6,87	0,001	2,36	1,08–5,16	0,031
Fibrose de AE	1,05	1,01–1,09	0,033	–	–	0,881
Volume do AE (indexado para ASC)	1,03	1,01–1,06	0,006	–	–	0,153

*FA: fibrilação atrial; ASC: área de superfície corporal; TAE_CE_: tecido adiposo epicárdico; AE: átrio esquerdo; RR: razão de risco; IC: intervalo de confiança.*

## Discussão

As principais conclusões deste estudo são essencialmente duas: 1) O tecido adiposo epicárdico e a fibrose do AE estão fracamente correlacionados; e 2) O tecido adiposo epicárdico parece ser um preditor mais poderoso de recidiva da FA do que a fibrose do AE. O tecido adiposo epicárdico demonstrou ser metabolicamente ativo, com atividade endócrina e parácrina.^6^ Especificamente, o secretoma da gordura epicárdica humana, mas não do tecido adiposo subcutâneo, tem efeitos pró-fibróticos no miocárdio atrial de ratos.^7^ O TAE também é conhecido por secretar ativina A, um membro da classe TGF-β capaz de induzir fibrose atrial.^6^ Um estudo recente também mostrou associação entre o TAE e a condução atrial lenta, maior fracionamento do eletrograma e aumento da fibrose atrial.^8^ A fibrose atrial induzida por gordura, portanto, parece ser um mecanismo explicativo razoável para a relação entre TAE e FA. Até o momento, as evidências de suporte para essa “hipótese fibrogênica” vieram de análises histológicas e bioquímicas de amostras obtidas em cirurgia cardíaca.^2^ Até onde sabemos, nosso estudo é a primeira avaliação in vivo da relação entre o volume do TAE e o volume de fibrose do AE em pacientes com FA. A fraca correlação que encontramos entre esses dois parâmetros não refuta uma conexão fisiopatológica, mas sugere que a fibrose do AE não é o único ou principal mecanismo pelo qual o TAE e a AF estão relacionados. O fato de o TAE estar mais fortemente associado à recidiva da FA do que a própria fibrose do AE corrobora a existência de outros mediadores mais importantes entre a adiposidade epicárdica e essa arritmia. Entre esses mediadores, podem se destacar ação pró-inflamatória de citocinas secretadas pelo TAE, incluindo proteína C reativa, interleucinas 1β, 6 e 8 e fator de necrose tumoral α, que podem ter efeitos arritmogênicos.^9 - 12^ A infiltração de gordura é outro mecanismo possível, com alguns estudos mostrando que o aumento do volume do TAE está associado à infiltração direta do miocárdio atrial,^13^ possivelmente causando prolongamento dos índices da onda P. Esse atraso na condução do tecido atrial pode ser um possível mecanismo para o início e para a manutenção da FA.^14^

No presente estudo, usamos uma modificação do método de TC proposto por Tran et al.^15^ para medir o TAE. Esse método usa uma medida de corte único do TAE no nível do tronco da coronária esquerda, produzindo resultados altamente correlacionados com o tecido adiposo epicárdico total.^15^ Esse método de quantificação do TAE foi escolhido por sua simplicidade e boa reprodutibilidade, mas deve-se ressaltar que atualmente não há consenso sobre a melhor metodologia para mensurar a gordura epicárdica, etapa que será fundamental para que esse parâmetro seja utilizado na prática clínica. Problema semelhante ocorre com a medição in vivo da fibrose do AE, em que é necessário o uso de protocolos padronizados para garantir a uniformidade de aquisição e processamento de imagens.^16^

### Limitações

Diversas limitações do presente estudo devem ser consideradas. Usamos uma amostra de conveniência de pacientes submetidos à ablação de FA, que pode não ser representativa da população global de FA. A recidiva da FA pode ser subnotificada, uma vez que o protocolo de seguimento não incluiu monitorização ecocardiográfica contínua. Por outro lado, episódios sintomáticos não documentados podem não representar uma verdadeira recidiva da FA e, portanto, resultar em uma superestimativa da recidiva. Além disso, não medimos a gordura epicárdica total: apenas uma pequena parte. Apesar dessas limitações, nossos achados podem contribuir para os esforços em curso para desvendar as ligações fisiopatológicas entre FA, TAE e fibrose do AE.

## Conclusão

A fraca correlação entre o TAE e a fibrose do AE sugere que a última não é o principal mecanismo pelo qual o TAE e a AF estão relacionados. O TAE esteve mais fortemente associada à recidiva da FA do que à fibrose do AE, corroborando ainda mais a existência de outros mediadores mais importantes entre o TAE e a FA.
