# Supplementary material for: Relação entre Gordura Epicárdica e Fibrilação Atrial Não Pode Ser Totalmente Explicada pela Fibrose Atrial Esquerda
Source: Arq Bras Cardiol. 2022 Jan 11;118(4):737–42. [Article in Portuguese] doi: 10.36660/abc.20201083 (PMC9007010; doi:10.36660/abc.20201083)
Supplement: Translation

# Translation: The Relationship between Epicardial Fat and Atrial Fibrillation Cannot Be Fully Explained by Left Atrial Fibrosis

**Authors:** Daniel Matos, António Miguel Ferreira, Pedro Freitas, Gustavo Rodrigues, João Carmo, Francisco Costa, João Abecasis, Pedro Carmo, Carla Saraiva, Diogo Cavaco, Francisco Morgado, Miguel Mendes, Pedro Adragao

**Affiliations:** 1 Portugal Centro Hospitalar de Lisboa Ocidental EPE Hospital de Santa Cruz, Lisboa – Portugal

**Keywords:** Atrial fibrillation, Atrial Fibrosis, Epicardial Fat, Pulmonary Vein Isolation

## Abstract

**Background:**

Epicardial adipose tissue (EAT) has been associated with atrial fibrillation (AF), but its pathophysiological mechanisms remain unclear.

**Objectives:**

To measure the correlation between EAT and left atrium (LA) fibrosis, and to assess their ability to predict relapse after pulmonary vein isolation (PVI).

**Methods:**

Patients with AF enrolled for a first PVI procedure underwent both cardiac computerized tomography (CT) and cardiac magnetic resonance (CMR) imaging within less than 48 hours. EAT_LM_was quantified on contrast-enhanced CT images at the level of the left main. LA fibrosis was quantified on isotropic 1.5 mm 3D delayed enhancement CMR. After pulmonary vein isolation (PVI), patients were followed up for AF relapse. Statistical significance was set at p<0.05.

**Results:**

Most of the 68 patients (46 men, age 61±12 years) had paroxysmal AF (71%, n=48). Patients had a median EAT_LM_volume of 2.4 cm^3^/m^2^(interquartile range [IQR] 1.6–3.2 cm^3^/m^2^), and a median amount of LA fibrosis of 8.9 g (IQR 5–15 g). The correlation between EAT_LM_and LA fibrosis was statistically significant but weak (Spearman’s R=0.40, p=0.001). During a median follow-up of 22 months (IQR 12–31), 31 patients (46%) had AF relapse. Multivariate analysis yielded two independent predictors of AF relapse: EAT_LM_(HR 2.05, 95% CI 1.51–2.79, p<0.001), and non-paroxysmal AF (HR 2.36, 95% CI 1.08–5.16, p=0.031).

**Conclusion:**

The weak correlation between EAT and LA suggests that LA fibrosis is not the main mechanism linking EAT and AF. EAT was more strongly associated with AF relapse than LA fibrosis, supporting the existence of other more important mediators of EAT and AF.

## Introduction

Epicardial adipose tissue (EAT) has recently been shown to be associated with the presence, severity, and relapse of atrial fibrillation (AF).^1^ Although the pathophysiological mechanisms underlying this association remain to be established, several hypotheses have been put forward, including direct adipocyte infiltration, oxidative stress, and the secretion of adipokines causing inflammation and fibrosis of atrial tissue.^1^ Establishing whether this relationship is causal, and ascertaining its underlying processes may prove useful to better understand AF and identify potential therapeutic targets. Thus far, the evidence linking EAT and atrial fibrosis has come mostly from histological and biochemical analyses of samples obtained from cardiac surgery,^2^ but both of these features can be assessed non-invasively. In this study, we aimed to measure the correlation between the volume of EAT and the amount of left atrium (LA) fibrosis assessed by non-invasive imaging, and to assess their ability to predict time to relapse after pulmonary vein isolation (PVI).

## Methods

### Study population

All consecutive patients with symptomatic drug-refractory AF undergoing cardiac computed tomography (CT) prior to percutaneous PVI at Hospital Santa Cruz (Carnaxide, Portugal) between November 2015 and December 2017 who underwent both cardiac computerized tomography (CT) and cardiac magnetic resonance (CMR) imaging within less than 48 hours were included in an observational registry used for this retrospective study. Patients with moderate or severe valvular heart disease, left atrial thrombus, abnormal thyroid function, or contraindication to anticoagulation were excluded. Atrial fibrillation was categorized as paroxysmal if self-terminated in less than 7 days, persistent if episodes lasted ≥7 days or required cardioversion, or long-standing persistent if AF was maintained for more than 12 months. This observational registry conforms to the ethical guidelines of the declaration of Helsinki and was approved by the institutional review board. All patients signed a an informed consent form.

### Cardiac CT and CMR protocols

All patients underwent cardiac CT and CMR imaging less than 72 hours before the ablation procedure for the assessment of pulmonary vein anatomy, measurement of LA volume, exclusion of thrombi and integration with electroanatomical mapping.

CT scans were performed on a dual-source 64-slice scanner (Somatom Definition^®^, Siemens Healthineers^®^, Erlangen, Germany) with injection of 90 mL nonionic contrast medium (400 mg I/mL iomeprol Bracco^®^) at a flow rate of 5 mL/s followed by 30–50 mL of saline solution. Scan parameters included detector collimation of 2x32x0.6 mm, slice acquisition of 64x0.6 mm, gantry rotation time of 330 ms, automatic exposure control, and tube potential of 100 kV (except if body mass index greater than 30 kg/m^2^and bodyweight over 90 kg, where 120 kV were used).

Prospective ECG tube current modulation was systematically used to minimize radiation exposure. Image reconstruction was performed with a slice thickness of 0.75 mm.

CMR images were acquired on a 1.5 T scanner (Magnetom Avanto^®^, Siemens Healthineers). The scan protocol included an isotropic 1.5 mm 3D inversion-recovery gradient-recalled-echo sequence with fat saturation and respiratory navigator, acquired 15 to 20 min after administration of 0.2 mmol/kg intravenous gadobutrol. Inversion time was chosen individually in order to nullify normal myocardium, using a TI scout sequence.

ECG gating was used to set the timing of image acquisition to late ventricular systole (LA diastole).

### Image Analysis

The CT quantification of EAT was performed semi-automatically on axial images using a TeraRecon Aquarius^®^Workstation (version 4.4.12, TeraRecon^®^, San Mateo, CA, USA). Four contiguous slices centered on the ostium of the left main (LM) were selected for analysis. The pericardium was manually traced in the first and last images, and automatically interpolated in the two middle slices, which were then checked for accuracy and adjusted if necessary. EAT_LM_volume was defined as the total volume of tissue within the pericardial sac in this 4-slice region of interest with attenuation values between -250 and -30 Hounsfield units.^1^ Left atrial volume was calculated by tracing the LA borders on CT images, excluding the pulmonary veins and the left atrial appendage.^3^

CMR post-processing for the quantification of LA fibrosis was performed with ADAS^®^software (version 2.3.3, Galgo Medical). LA wall contours were drawn manually, excluding the mitral valve and pulmonary veins from the analysis. The signal intensity of the LA wall was normalized using an image intensity ratio (IIR) calculated as the ratio between the signal intensity of each pixel and the mean blood pool intensity. IIR >1.20 was considered to represent LA fibrosis.^4^ Quantifications of LA fibrosis and EAT were performed only after the ablation procedure (without knowing its outcome). Examples of EAT and LA fibrosis quantifications are presented in Figure 1 .

**Figure 1:** – *Epicardial fat (A) measured with computed tomography and left atrium fibrosis (B) measured with cardiac magnetic resonance.*

### Pulmonary vein isolation protocol

Pulmonary vein isolation was guided by electroanatomical mapping, using either NavX^®^(St Jude Medical^®^, St Paul, MN, USA) or CARTO^®^(Biosense Webster^®^, Diamond Bar, CA, USA) systems. The right femoral vein was used as the preferred vascular access, through which three catheter electrodes were introduced: (i) a decapolar catheter, advanced through the coronary sinus; (ii) a variable circular mapping catheter, placed in the pulmonary veins; and (iii) an irrigated contact force-sensing ablation catheter. Left atrial access was established by transseptal puncture. Radiofrequency ablation was performed more than 5 mm from the pulmonary vein ostia, with continuous lesions enclosing the left and right pairs of pulmonary veins.^5^

Treatment was considered successful if bidirectional block was achieved. When required, electrical cardioversion was performed at the end of the procedure. Oral anticoagulation was resumed 6 hours after the ablation, maintained for 6 months, and then withdrawn or continued according to CHA_2_DS_2_-VASc criteria. Class I/III antiarrhythmic drugs were generally maintained in all of the patients for the first 3 months after the procedure, then withdrawn if there was no AF relapse. A proton pump inhibitor was prescribed for the first month after the ablation.

### Study endpoint and patient follow-up

The study endpoint was AF relapse, defined as symptomatic or documented AF and/or other atrial arrhythmias, after a 3-month blanking period. Symptomatic AF was defined as the presence of symptoms likely due to AF episodes. Documented AF was defined by the presence of at least one episode of AF lasting more than 30 seconds in an ECG, 24-hour Holter monitoring, or event-loop recording. The follow-up protocol consisted of outpatient visits with 12-lead ECG and 24-h Holter monitoring at the assistant physicians’ discretion (typically at 6 and 12 months, and yearly thereafter). If the clinical records were insufficient, a structured telephone interview was conducted. Patients kept on antiarrhythmic drugs after the third month of follow-up were not considered as failed ablation.

### Statistical analysis

Normally and non-normally distributed continuous variables were expressed as mean±standard deviation and median and interquartile range, respectively, while categorical variables were expressed as frequencies and percentages. Statistical significance was set at p<0.05. Shapiro-Wilk test was employed to assess the population’s normality. Unpaired Student’s t-test was used to assess statistically significant differences between normally distributed continuous variables, while Mann-Whitney U test was used for non-normally distributed continuous variables. Chi-square test was used to analyze categorical variables. Spearman’s correlation coefficient was used for gauging the correlation between EAT_LM_volume and LA fibrosis. Univariate proportional-hazards Cox regression was used to identify predictors of time to AF relapse. Variables with p-value ≤0.10 in univariate analysis were selected for a multivariate Cox regression model and considered statistically significant if p<0.05. A 95% confidence level was used in our statistical analysis. Statistical analysis was performed using the Statistical Package for Social Sciences (SPSS) version 22.0 (SPSS Inc., Chicago, IL).

## Results

The baseline characteristics of the study population are presented in Table 1 . Overall, patients had a median EAT_LM_volume of 2.4 cm^3^/m^2^(interquartile range [IQR] 1.6–3.2 cm^3^/m^2^), and a median estimated amount of LA fibrosis of 8.9 g (IQR 5–15 g), corresponding to 8% (IQR 5–11%) of the total LA wall mass.

**Table 1:** – Baseline characteristics of the study population

Baseline characteristics	Total (n=68)	Without AF relapse (n=37)	With AF relapse (n=31)	p-value
Age, years	61±12	61±11	61±12	0.968
Male sex, n (%)	46 (67.6)	22	24	0.312
Weight, kg	81±13	79±12	82±13	0.097
Body mass index, kg/m^2^	28±4	27±4	29±4	0.091
Type of AF				0.003
paroxysmal, n (%)	48 (70.6)	32	16	
non-paroxysmal, n (%)	20 (29.4)	5	15	
Hypertension, n (%)	41 (60.3)	21	20	0.621
Diabetes, n (%)	6 (8.8)	5	1	0.209
Active smoking, n (%)	4 (5.9)	1	3	0.304
LV systolic dysfunction, n (%)	0 (0)	0	0	1.000
Known CAD, n (%)	6 (8.8)	4	2	0.366
CHA_2_DS_2_-VASc, median (IQR)	2 (1–3)	2 (1–3)	2 (1–3)	0.578
LA volume on cardiac CT, mL/m^2^	56±15	52±12	60±17	0.025
EAT_LM_volume, mL/m^2^	2.4±1.2	1.9±0.7	3.1±1.2	<0.001
LA fibrosis, g, median (IQR)	8.9 (5–15)	6.7 (4–13)	11.2 (6–17)	0.049
LA fibrosis, % of LA mass, median (IQR)	7.5 (5–11)	6.9 (4–11)	8.8 (6–12)	0.170

*AF: atrial fibrillation; LV: left ventricle; LA: left atrium; CT: computed tomography; EAT_LM_: epicardial adipose tissue.*

The correlation between EAT_LM_and LA fibrosis was statistically significant but weak (Spearman’s correlation coefficient = 0.40, p=0.001) – Figure 2 .

**Figure 2:** – *Left atrial fibrosis and epicardial fat correlation graphic. EAT_LM_: epicardial adipose tissue.*

During a median follow-up of 22 months (IQR 12–31), 31 patients (46%) had AF relapse. Patients who had AF relapse were more likely to have non-paroxysmal AF, and had higher LA volumes, higher amounts of EAT_LM_and LA fibrosis. On assessing the time to AF relapse, these four predictors were identified in univariate Cox regression. Multivariate analysis yielded two independent predictors of time to AF relapse: EAT_LM_and non-paroxysmal AF ( Table 2 ).

**Table 2:** – Univariate and multivariate Cox regression of AF-relapse predictors

Predictors of AF relapse	Univariate Analysis			Multivariate Analysis		
	HR	95% CI	p-value	HR	95% CI	p-value
EAT_LM_	2.19	1.65–2.91	<0.001	2.05	1.51–2.79	<0.001
Non-paroxysmal AF	3.36	1.64–6.87	0.001	2.36	1.08–5.16	0.031
LA fibrosis	1.05	1.01–1.09	0.033	–	–	0.881
LA volume (indexed to BSA)	1.03	1.01–1.06	0.006	–	–	0.153

*AF: atrial fibrillation; BSA: body surface area; EAT_LM_: epicardial adipose tissue; LA: left atrium; HR: hazard ratio; CI: confidence interval.*

## Discussion

The main findings of this study are essentially twofold: 1) epicardial adipose tissue and LA fibrosis are weakly correlated; and 2) epicardial adipose tissue seems to be a more powerful predictor of AF relapse than LA fibrosis. Epicardial adipose tissue has been shown to be metabolically active, with endocrine and paracrine activity.^6^ Specifically, the secretome from human epicardial fat, but not from subcutaneous adipose tissue, has pro-fibrotic effects on the atrial myocardium of rats.^7^ EAT is also known to secrete activin A, a member of the TGF-β class capable of inducing atrial fibrosis.^6^ A recent study also showed an association between EAT and slow atrial conduction, greater electrogram fractionation and increased atrial fibrosis.^8^ Fat-induced atrial fibrosis would therefore seem to be a reasonable explanatory mechanism for the link between EAT and AF. Thus far, supporting evidence for this “fibrogenic hypothesis” has come from histological and biochemical analyses of samples obtained from cardiac surgery.^2^ To the best of our knowledge, our study is the first *in vivo* assessment of the relationship between EAT volume and the amount of LA fibrosis in patients with AF. The weak correlation we found between these two parameters does not disprove a pathophysiological connection but suggests that LA fibrosis is not the sole or main mechanism by which EAT and AF are linked. The fact that EAT was more strongly associated with AF relapse than LA fibrosis itself further supports the existence of other more important mediators between epicardial adiposity and this arrhythmia. These may include pro-inflammatory action of cytokines secreted by EAT including C-reactive protein, interleukins 1β, 6 and 8 and tumor necrosis factor α, which may have arrhythmogenic effects.^9 - 12^ Fatty infiltration is another possible mechanism, with some studies showing that increased EAT volume is associated with direct infiltration of the atrial myocardium,^13^ possibly causing prolongation of P-wave indices. This delay in atrial tissue conduction may be a potential mechanism for initiation and maintenance of AF.^14^

For this study, we used a modification of the CT method proposed by Tran et al.^15^ to measure EAT. This method uses a single slice measure of EAT at the level of the left main coronary artery, yielding results that are highly correlated with total epicardial adipose tissue.^15^ This EAT quantification method was selected for its simplicity and good reproducibility, but it should be noted that there is currently no consensus on the best methodology to measure epicardial fat, a step that will be crucial if this parameter is to be used in clinical practice. A similar problem occurs with the *in vivo* measurement of LA fibrosis, where the use of standardized protocols is necessary to ensure uniformity of image acquisition and processing.^16^

### Limitations

Several limitations of this study should be acknowledged. We used a convenience sample of patients undergoing AF ablation, who may not be representative of the global AF population. AF relapse may be underreported, since the follow-up protocol did not include continuous ECG monitoring. On the other hand, symptomatic undocumented episodes may not represent true AF relapse and thus result in overestimation of relapse. Also, we did not measure total epicardial fat, but only a limited portion of it. Despite these limitations, our findings may contribute to the ongoing efforts to uncover the pathophysiological links between AF, EAT and LA fibrosis.

## Conclusion

The weak correlation between EAT and LA fibrosis suggests that the latter is not the main mechanism by which EAT and AF are linked. EAT was more strongly associated with AF relapse than LA fibrosis, further supporting the existence of other more important mediators between EAT and AF.
